# Plasma mRNA expression levels of BRCA1 and TS as potential predictive biomarkers for chemotherapy in gastric cancer

**DOI:** 10.1186/s12967-014-0355-2

**Published:** 2014-12-14

**Authors:** Jie Shen, Jia Wei, Wenxian Guan, Hao Wang, Yitao Ding, Xiaoping Qian, Lixia Yu, Zhengyun Zou, Li Xie, Carlota Costa, Trever Bivona, Rafael Rosell, Baorui Liu

**Affiliations:** The Comprehensive Cancer Centre of Drum Tower Hospital, Medical School of Nanjing University, Clinical Cancer Institute of Nanjing University, 321 Zhongshan Rd, Nanjing, 210008 China; Drum Tower Hospital, Medical School of Nanjing University, Nanjing, China; Pangaea Biotech, Dexeus University Institute, Barcelona, Spain; Department of Medicine, University of California, San Francisco, CA USA; Catalan Institute of Oncology, Medical Oncology Service, Hospital Germans Trias i, Pujol, Ctra Canyet s/n, Badalona, 08916 Spain

**Keywords:** Plasma mRNA, Gastric cancer, Chemosensitivity, Predictive biomarker, HDRA

## Abstract

**Objective:**

Personalized chemotherapy based on predictive biomarkers can maximize efficacy. However, tumor tissue obtained at the time of initial diagnosis will not reflect genetic alterations observed at the time of disease progression. We have examined whether plasma mRNA levels can be a surrogate for tumor levels in predicting chemosensitivity.

**Methods:**

In 150 gastric cancer patients, mRNA levels of BRCA1 and TS were assessed in plasma and paired tumor tissue. The Mann-Whitney U-test was used to compare mRNA expression levels between tumor samples exhibiting *in vitro* sensitivity or resistance to docetaxel and pemetrexed. All statistical tests were two-sided.

**Results:**

There were significant correlations between plasma and tumor mRNA levels of BRCA1 (rho = 0.696, P < 0.001) and TS (rho = 0.620, P < 0.001). BRCA1 levels in plasma (docetaxel-sensitive: 1.25; docetaxel-resistant: 0.50, *P* < 0.001) and tumor (docetaxel-sensitive: 8.81; docetaxel-resistant: 4.88, *P* < 0.001) were positively associated with docetaxel sensitivity. TS levels in plasma (pemetrexed-sensitive: 0.90; pemetrexed-resistant: 1.82, *P* < 0.001) and tumor (pemetrexed-sensitive: 6.56; pemetrexed-resistant: 16.69, *P* < 0.001) were negatively associated with pemetrexed sensitivity.

**Conclusions:**

Plasma mRNA expression levels mirror those in the tumor and may have a promising role as potential predictive biomarkers for chemotherapy.

**Electronic supplementary material:**

The online version of this article (doi:10.1186/s12967-014-0355-2) contains supplementary material, which is available to authorized users.

## Introduction

The standard first-line chemotherapy regimen for locally advanced or metastatic gastric cancer is cisplatin or oxaliplatin combined with other drugs, including docetaxel and pemetrexed [1,2]. However, median survival remains meager – around one year. No standard approach for second-line therapy exists, although docetaxel has shown some activity [3]. Personalized chemotherapy based on the mRNA expression of predictive biomarkers could help to maximize treatment efficacy and prolong survival in these patients [3-8].

Breast cancer susceptibility gene 1 (BRCA1), an essential component in multiple DNA damage repair pathways and pathways involved in cellular responses to microtubule damage, is considered to be a differential modulator of survival with cisplatin and taxanes. Preclinical and clinical studies have reported that BRCA1 level of tumor is associated with cisplatin and taxanes chemosensitivity. Tumor with high expression of BRCA1 is 800-to-more than 1000-fold sensitive to docetaxel, but 10-1000-fold resistant to cisplatin [4]. We have previously observed that BRCA1 mRNA levels in effusions were negatively associated with platinum sensitivity but positively associated with docetaxel sensitivity in gastric cancer patients [5]. These findings were validated in gastric patients receiving second-line docetaxel: median survival was 9.5 months in those with low BRCA1 levels in primary tumor, 19.1 in those with intermediate levels, and 25.8 months in those with high levels (*P* = 0.006) [3]. Pemetrexed is a new kind of water-soluble quinazoline folate analogue, acts as a direct and specific thymidylate synthase (TS) inhibitor. Low expression of thymidylate synthase (TS) has been reported to be associated with response to pemetrexed-based therapy in lung cancer [7,8].

Tumor tissue is the major source for biomarker examination at present. However, tumor tissue may at times be insufficient for gene expression analysis. Moreover, tumor tissue obtained at the time of initial diagnosis may not reflect genetic alterations observed at the time of disease progression [9], since metastatic and primary tumors from the same patient can vary at genomic, epigenetic and transcriptomic levels [9-13]. Furthermore, initial chemotherapy may itself alter gene expression levels [14,15]. Circulating tumor cell-free nucleic acids could thus be a useful, non-invasive tool for tracking changes during the course of treatment [10], obviating the need for a re-biopsy at the time of determining the best treatment. In addition, it can be a useful surrogate when only a small amount of tumor tissue is available. Our previous experience in lung cancer demonstrated that epidermal growth factor receptor (EGFR) mutations in serum could be a good predictive marker for lung cancer patients receiving EGFR tyrosine kinase inhibitors [16]. In addition, plasma TS mRNA can be detected non-invasively in blood before surgery and the expression level of plasma TS had great potential as predictive biomarkers for raltitrexed sensitivity in gastric cancer [17]. To date, however, little is known about the potential use of plasma mRNA for predicting chemosensitivity of docetaxel and pemetrexed. Moreover, although RNA released into circulation is stable [10,18], with currently available methods, only a small amount of mRNA can be obtained from plasma or serum, which limits both the efficacy of mRNA extraction and the consistency of mRNA assays [10].

In the present study, we adopted a practical and convenient method for plasma mRNA detection, based on RNA purification and quantitative RT-PCR (qRT-PCR), and used this method to examine whether plasma mRNA levels can be a surrogate for tumor levels in predicting chemosensitivity – first in a pilot study of 40 patients and then in a total cohort of 150 patients.

## Materials and methods

### Patients

From October 2010 to July 2011, 150 freshly-removed gastric tumors and paired blood samples from the same patients were obtained from the Department of Oncology and General Surgery, Drum Tower Hospital, Nanjing, China. All the patients have not received any chemotherapy before surgery. The fresh gastric tumors and blood samples were kept at 4°C and sent to the laboratory within 15 minutes of collection.

The study was approved by the Institutional Ethics Review Board of Drum Tower Hospital affiliated to the Medical School of Nanjing University, and written informed consent was provided by all patients. All animal experiments were performed in accordance with the Chinese Coordinating Committee on Cancer Research Regulations for the Welfare of Animals and the Animal Protection Law. All analyses, including drug sensitivity testing, gene expression analysis and animal experiments, were carried out by different investigators acting individually without knowledge of the results of the other analyses.

### Study design

**Figure 1:**
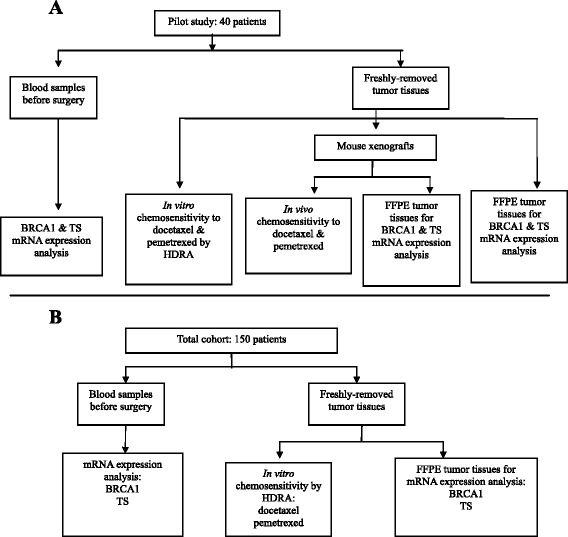
**Flow chart showing patient disposition and experiments performed.** Pilot study in 40 patients **(A)** and total cohort of 150 gastric cancer patients **(B)**.

We first carried out a pilot study in 40 patients (Figure 1A). For each tumor sample, sensitivity to docetaxel and pemetrexed was examined *in vitro* by histoculture drug response assay (HDRA) [19] and *in vivo* by xenografts in immunodeficient mouse models [20] (Additional file 1: Figure S1, A-B available online). In addition, both the mouse xenografts and the original fresh tumor samples were formalin-fixed and paraffin-embedded (FFPE) for the analysis of BRCA1 and TS mRNA expression. BRCA1 and TS expression was also analyzed in the paired blood samples.

Based on the findings of the pilot study, we undertook to validate our findings in the total cohort of 150 gastric cancer patients (Figure 1B). In this cohort, the same procedures were carried out, with the exception of the *in vivo* testing of chemosensitivity. The mRNA levels in plasma and FFPE tumor tissues of BRCA1 and TS were assessed and the Mann-Whitney U-test was used to compare mRNA expression levels between tumor samples exhibiting *in vitro* sensitivity or resistance to docetaxel and pemetrexed.

For complete details of all methodology, see Additional file 1, available online.

### *In vitro* chemosensitivity in the pilot study and the total cohort

Fresh tumor tissues were sent to the laboratory in 4°C Hanks’ balanced salt solution with 1% penicillin/streptomycin. Specimens were washed with Hanks’ balanced salt solution twice and minced into small pieces of approximately 10 mg, which were then placed on prepared collagen surfaces in 24-well microplates. Plates were incubated for 7 days at 37°C in the presence of drugs dissolved with RPMI 1640 medium containing 20% fetal calf serum and kept in a humidified atmosphere containing 95% air–5% CO_2_. Concentrations of drugs were 100 μg/ml for docetaxel [21], and 400 μg/ml for pemetrexed [7]. HDRA procedures were performed as reported by Furukawa and colleagues [19] with slight modifications. The inhibition rate was calculated by using the following formula: inhibition rate (%) = (1-T/C) x 100, where T is the mean absorbance of treated tumor/weight and C is the mean absorbance of control tumor/weight. All samples were classified into sensitive and resistant groups using the minimum *P* value method as modified by Lausen and Schumacher [22].

### *In vivo* chemosensitivity in the pilot study

Each freshly-removed surgical tumor tissue was cut into pieces of 3 × 3 × 3mm^3^, which were transplanted within 30 minutes to 9-18 athymic immunodeficient mice, termed a “panel”. In each panel, when the tumor grew to a size of 50-100 mm^3^, mice with xenografts were randomized to treatment with docetaxel 15 mg/kg/d, ip or pemetrexed 20 mg/kg/d, ip or no treatment (control). Individual tumor volumes (V) were calculated by the formula “V = (length × width × width) / 2” and compared to the values at the start of treatment to obtain the relative tumor volume. Median treated-to-control values of relative tumor volume were termed “inhibition rate *in vivo*” and were used to assess sensitivity to docetaxel or pemetrexed.

### mRNA expression in the pilot study and the total cohort

Two milliliters of blood were collected in EDTA tubes before surgery, kept at 4°C and sent to the laboratory within 15 minutes. Total RNA from plasma was extracted with TRIzol LS and chloroform, and purified with the PureLink RNA Mini Kit (Ambion, Carlsbad, CA, USA). Total RNA from FFPE tumor tissues was extracted in accordance with a proprietary procedure (European patent number EP1945764-B1). After RNA extraction and purification, M-MLV Reverse Transcriptase Kit (Invitrogen, Carlsbad, CA, USA) was used to generate cDNA for Q-PCR to detect the expression of β-actin (ACTB – used as endogenous control) and the genes examined. Each batch of reaction included a positive control from commercial human lung and liver RNA (Stratagene, La Jolla, CA, USA) as calibrators and negative controls without RNA and reverse transcriptase. Template cDNA was amplified with specific primers and probes using Taqman Universal Master Mix (Applied Biosystems, Foster City, CA, USA). Relative gene expression quantifications were calculated according to the comparative Ct method. Final results were determined by the formula mRNA expression level = 2^-△△Ct^ [6,23] and were analyzed with the Stratagene software.

### Reproducibility and stability of the gene expression assay in plasma

The reproducibility of the gene expression assay was tested simultaneously with two methods. For the first method, blood samples from five patients were collected from the first day to the fifth day before surgery at the same time each day. During this period, patients received neither chemotherapy nor special treatment. Gene expression levels (high/intermediate vs low) were determined for each sample, and the percentage of identical results was calculated for each patient. For the second method, blood was drawn once each from an additional ten patients, and each sample was divided in five aliquots. Gene expression levels (high/intermediate vs low) were determined for each aliquot, and the percentage of identical results was calculated for each patient. The reproducibility rate was defined as the average of all 15 percentages (5 patients from method one and 10 patients from method two).

In order to test the stability of the blood samples, different plasma aliquots of the same sample were prepared under distinct conditions. Aliquots were incubated at room temperature or on ice for 30 min, 1 h, 1.5 h or 2 h. qRT-PCR was then performed, and raw ΔCt values of different gene were compared with the standard protocol (ΔCt = Ct _gene_-Ct _ACTB_ ).

### Statistical analyses

The Mann-Whitney U-test and the Kruskal-Wallis test were used to test associations between mRNA levels and clinical characteristics and between sensitivity to each chemotherapeutic agent and clinical characteristics. The Spearman rank correlation test was used to assess the correlation between *in vivo* and *in vitro* inhibition rates and between plasma and tumor mRNA expression levels. The Mann-Whitney U-test was used to compare mRNA expression levels between tumor samples exhibiting *in vitro* sensitivity or resistance to docetaxel and pemetrexed. Receiver operating characteristics (ROC) curves were constructed to assess sensitivity, specificity, and respective areas under the curves (AUCs) with their 95% confidence intervals (CIs). We investigated the optimized cut-off value for prediction by maximizing the sum of sensitivity and specificity and minimizing the overall error (square root of the sum [1-sensitivity]^2^ + [1-specifi city]^2^), and by minimizing the distance of the cut-off value to the top-left corner of the ROC curve [24]. All statistical tests were two-sided, and significance was set at *P* < 0.05 (two-sided). All analyses were performed using SPSS, version 16.0.

## Results

Characteristics of all patients are shown in Table 1. The majority of patients were males (73.3%), and the predominant histology was adenocarcinoma. Ninety-four (62.7%) patients had stage III disease. Lymph node metastases were present in 115 (76.7%) patients.

**Table 1:** **Patient characteristics**

**Characteristic**	**Pilot study**	Total cohort*
	**N = 40**	**N = 150**
	**N (%)**	**N (%)**
**Age, yr, median (range)**	63 (40-83)	64 (29-84)
**Sex**		
Male	31 (77.5)	110 (73.3)
Female	9 (22.5)	40 (26.7)
**Tumor site**		
Distal stomach	12 (30.0)	57 (34.0)
Proximal stomach	19 (47.5)	62 (41.3)
Whole stomach	9 (22.5)	37 (24.7)
**Stage**		
I	4 (10.0)	17 (11.3)
II	9 (22.5)	34 (22.7)
III	25 (62.5)	94 (62.7)
IV	2 (5.0)	5 (3.3)
**Histological grade**		
1	0	3 (2.0)
2	8 (20.0)	31 (20.7)
3	22 (55.0)	71 (47.3)
Mixed 1–2	0	2 (1.3)
Mixed 2–3	10 (25.0)	43 (28.7)
**Lymph node metastasis**		
No	9 (22.5)	35 (23.3)
Yes	31 (77.5)	115 (76.7)

*The total cohort consisted of the original 40 patients included in the pilot study plus an additional 110 patients.

The reproducibility of the plasma mRNA detection was 92% for BRCA1, and 89% for TS. The mRNA expression levels were stable after room temperature or ice incubation for 30 min, 1h, 1.5 h and 2 h (Additional file 1: Figure S2, available online).

### Pilot study

Sensitivity to docetaxel and pemetrexed was successfully assessed in the 40 fresh tumor samples by HDRA. Eleven panels of immunodeficient mice (9-18 mice per panel, 148 mice in total) with human-derived xenografts were successfully established from all 40 surgical specimens for *in vivo* analysis of sensitivity. The Spearman rank correlation test showed a significant correlation between *in vitro* and *in vivo* inhibition rates for docetaxel (rho = 0.900, *P* < 0.001) and pemetrexed (rho = 0.836, *P* < 0.001) (Additional file 1: Figure S3, A-B, available online).

BRCA1 mRNA levels were successfully assessed in all 40 tumor samples, in 32 paired blood samples, and in 10 panels of mouse models, while TS mRNA levels were successfully assessed in all 40 tumor samples, all 40 blood samples, and in 9 panels of mouse models. The Spearman rank correlation test showed a correlation between plasma and tumor mRNA levels of BRCA1 (rho = 0.647, *P* < 0.001) and between plasma and tumor levels of TS (rho = 0.615, *P* < 0.001) (Additional file 1: Figure S4, A-B, available online). Both plasma and tumor BRCA1 levels were correlated with docetaxel sensitivity (rho = 0.492, *P* = 0.004 and rho = 0.527, *P* < 0.001, respectively), and both plasma and tumor TS levels correlated with pemetrexed resistance (rho = -0.627, *P* < 0.001 and rho = -0.443, *P* = 0.004, respectively, Spearman rank correlation test) (Additional file 1: Figure S5, A-D, available online). BRCA1 mRNA levels were higher in the docetaxel-sensitive than in the docetaxel-resistant mouse models (11.26 vs 2.63; *P* < 0.001), while TS levels were higher in the pemetrexed-resistant than in the pemetrexed-sensitive mice (3.34 vs 7.27; *P* = 0.013, Mann-Whitney U-test) (Additional file 1: Figure S6, A-B, available online).

### Total cohort

BRCA1 and TS mRNA expression was successfully assessed in tumor tissues from all 150 patients. The detection rate in plasma were 82.00% for BRCA1 and 90.67% for TS. The mean mRNA levels in plasma and tumor are shown in Additional file 1: Table S1, available online. There was no significant association between clinical characteristics and the mRNA expression levels of BRCA1 or TS (P > 0.05).

**Figure 2:**
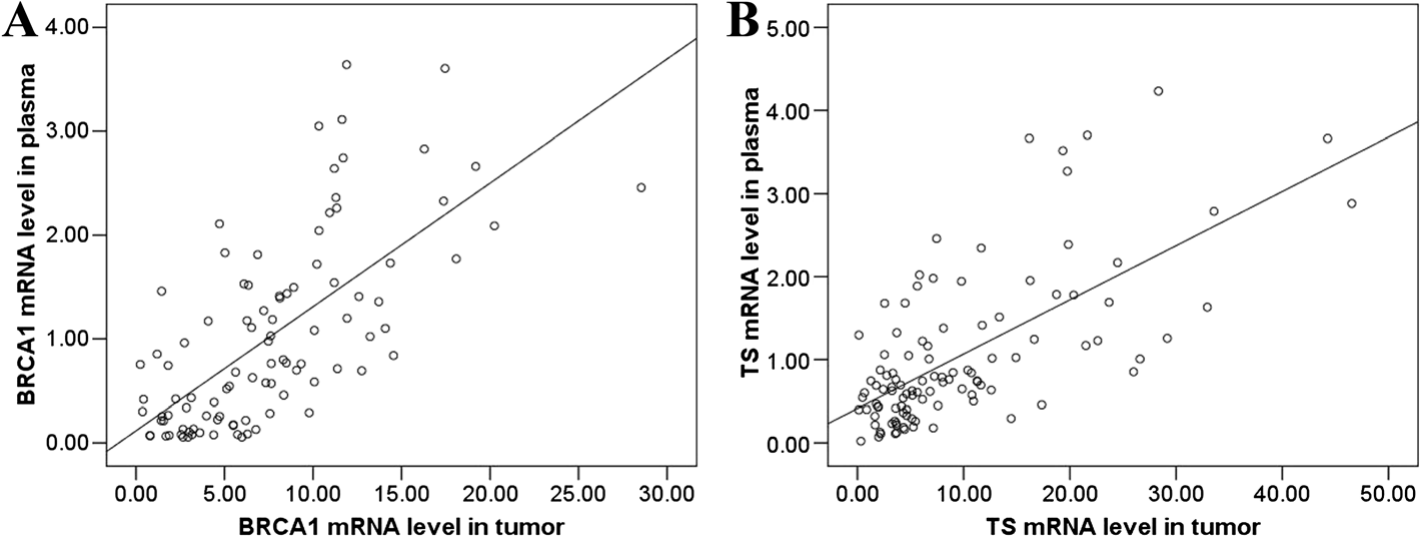
**Correlations between plasma and tumor mRNA expression levels of (A) BRCA1 and (B) TS in 150 gastric cancer patients.** There was a significant correlation between plasma and tumor mRNA expression levels of both genes (*P* < 0.001, respectively).

There was a positive correlation between plasma and tumor mRNA levels of BRCA1 (rho = 0.696, *P* < 0.001) and TS (rho = 0.620, *P* < 0.001, Spearman rank correlation test) (Figure 2A-B). No differences in baseline characteristics were observed according to plasma mRNA levels, tumor mRNA levels, or *in vitro* chemosensitivity.

**Figure 3:**
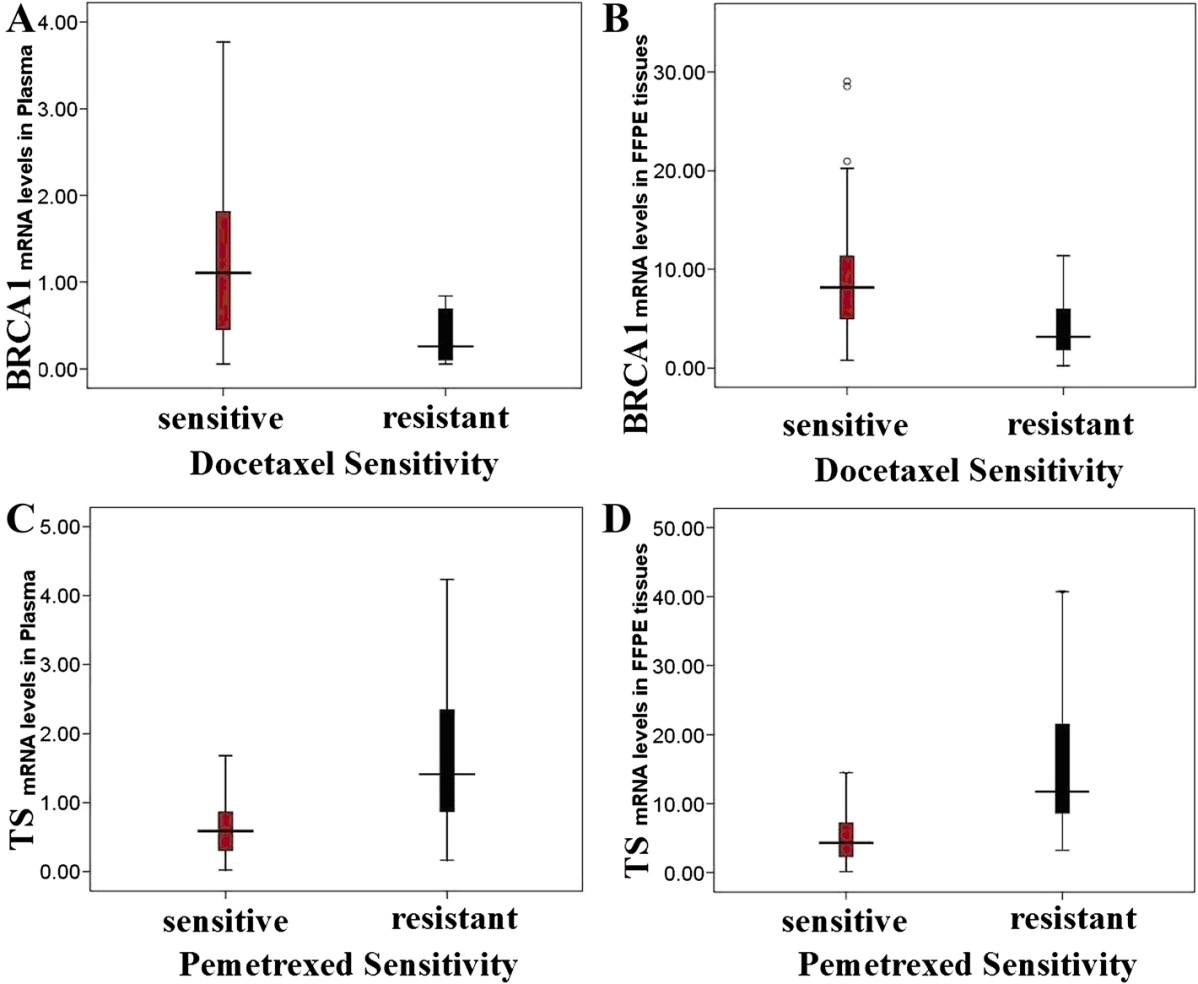
***In vitro*** **chemosensitivity associated with plasma and tumor expression levels of BRCA1and TS in 150 gastric cancer patients.** BRCA1 levels in plasma **(A)** and tumor **(B)** were higher in the docetaxel-sensitive than in the docetaxel -resistant group. TS levels in plasma **(C)** and tumor **(D)** were lower in the pemetrexed-sensitive than in the pemetrexed-resistant group. The lines inside the boxes denoted the medians. The whiskers of box plots: SE, 95% CI.

BRCA1 levels in plasma (docetaxel-sensitive: 1.25; docetaxel-resistant: 0.50, *P* < 0.001) and tumor (docetaxel-sensitive: 8.81; docetaxel-resistant: 4.88, *P* < 0.001) were positively associated with docetaxel sensitivity. BRCA1 levels in plasma and tumor were higher in the docetaxel-sensitive than in the docetaxel -resistant group. TS levels in plasma (pemetrexed-sensitive: 0.90; pemetrexed-resistant: 1.82, *P* < 0.001) and tumor (pemetrexed-sensitive: 6.56; pemetrexed-resistant: 16.69, *P* < 0.001) were negatively associated with pemetrexed sensitivity. TS levels in plasma and tumor were lower in the pemetrexed-sensitive than in the pemetrexed-resistant group. (Figure 3A-D, Mann-Whitney U-test).

**Figure 4:**
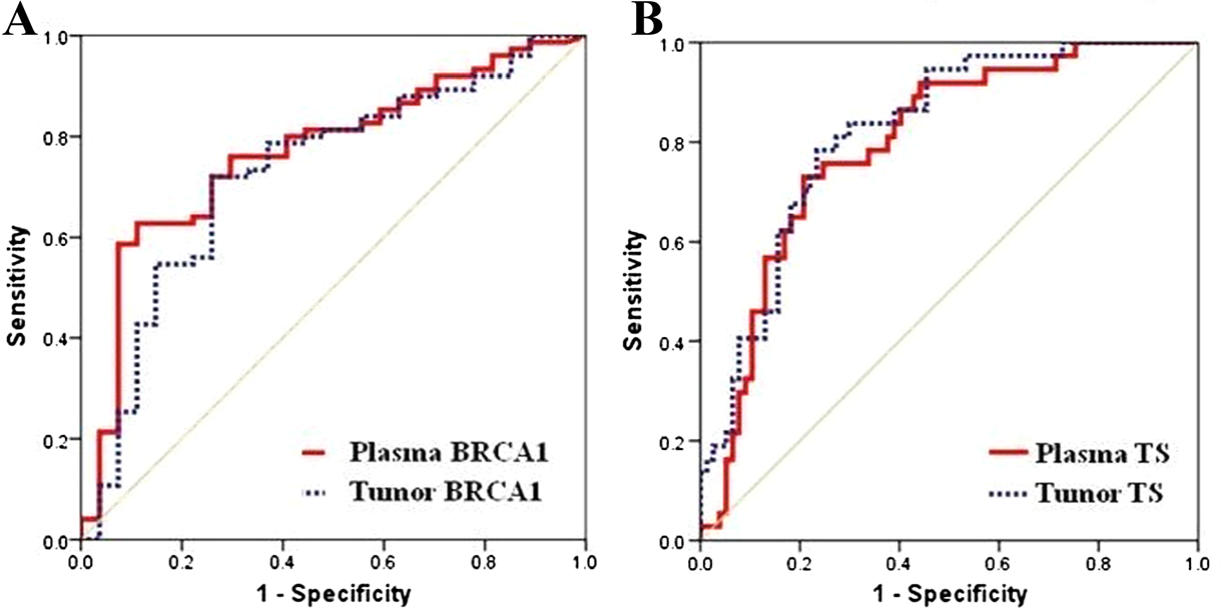
**ROC curves showing the sensitivity and specificity of plasma and tumor gene expression in predicting chemosensitivity. (A)** BRCA1 and docetaxel (n = 123); **(B)** TS and pemetrexed (n = 136).

Sensitivity in predicting chemosensitivity ranged from 63% to 71% for plasma and from 70% to 78% for tumor mRNA. Specificity ranged from 78% to 90% for plasma and 79% for tumor mRNA (Table 2, Figure 4).

**Table 2:** **Sensitivity and specificity of plasma and tumor gene expression levels for the prediction of chemosensitivity**

**Gene expression**	**Chemotherapeutic agents**	**N**	**Optimal cut-off**	**Sensitivity**	**Specificity**	**AUC (95% CI)**	***P***
**Plasma**							
BRCA1	Docetaxel	123	0.76	63%	90%	0.77 (0.67-0.87)	<0.001
TS	Pemetrexed	136	1.01	71%	78%	0.78 (0.69-0.87)	<0.001
**Tumor**							
BRCA1	Docetaxel	123	6.09	70%	79%	0.75 (0.66-0.84)	<0.001
TS	Pemetrexed	136	8.08	78%	79%	0.83 (0.76-0.90)	<0.001

## Discussion

In the present study, BRCA1 and TS mRNA levels in plasma were correlated with levels in paired FFPE tumor tissue. BRCA1 and TS mRNA levels in both plasma and tumor were associated with *in vitro* chemosensitivity of freshly explanted gastric cancer. Moreover, plasma mRNA levels showed considerable sensitivity and specificity in predicting chemosensitivity. Taken together, these findings indicate that plasma mRNA mirrors tumor mRNA and may prove a useful tool for predicting chemosensitivity.

The method we established to detect plasma mRNA, based on RNA enrichment by purification column, enables more accurate, sensitive and reproducible detection, easier handling and faster results [10,25], making it feasible for routine gene expression analysis in daily clinical practice [25]. However, the contamination of mRNA by non-tumor sources, such as activated lymphocytes, stromal cells or circulating tumor cells, cannot be ruled out [10,26,27] and may partially explain why the correlation between plasma and tumor expression was less than perfect*.* Moreover, tumor heterogeneity may account for some variability as well. Specificity ranged from 78% to 90% for plasma while 79% for tumor. The less perfect specificity in tumor may well be due to the heterogeneity of the tumor tissue itself, which lends further support to the use of plasma mRNA for predicting sensitivity.

Recent studies have examined the cell-free mRNA expression of several genes, including TS [28], β-catenin [29], hTERT [26,30,31], CK19 [32,33], MUC1 [34], CXCR4 [35], Bmi-1 [35], Her-2 [36] and DKK1 [24], these studies have focused on the use of cell-free mRNA for early diagnosis, tumor staging and disease monitoring. In contrast, to the best of our knowledge, the present study is the first to examine the potential role of plasma mRNA for predicting docetaxel and pemetrexed chemosensitivity of solid tumors. Our findings suggest a promising role for plasma mRNA expression as a predictive biomarker in gastric cancer and provide evidence for the use of plasma mRNA in selecting chemotherapy for patients without the need for re-biopsy at the time of disease progression. In addition, plasma mRNA may also prove useful for monitoring patients being treated in the neoadjuvant or adjuvant setting. The treatment plan for these patients could be generated according to their mRNA chemosensitivity profile. For example, gastric cancer patients with low levels of plasma TS mRNA can be treated with pemetrexed-based chemotherapy, while those with high levels of plasma BRCA1 could benefit from treatment with docetaxel.

Nonetheless, we should note that our study may have some limitations. The current study is based on patients, who are therapy-naïve. By collecting their plasma before surgery and tumors right after surgery, we could demonstrated the plasma mRNA value for monitoring patients being treated in the neoadjuvant or adjuvant setting. Then, the detection sensitivity and value of picking up circulating mRNA from early metastases especially for patients reserving second line chemotherapy is still unclear. Now we are also carrying out further study on patients with metastases and cannot receiving surgery or to receive second line chemotherapy. We will have the latest results in the near future which will contain the content mentioned above.

## Conclusion

In conclusion, plasma mRNA expression levels of BRCA1 and TS could mirror those in the tumor and may have a promising role as potential predictive biomarkers for docetaxel and peretrexed in gastric cancer. These findings are preliminary and only suggestive at this point, and each of these paired biomarker-chemotherapy hypotheses needs to be validated before being used in routine daily clinical practice. A clinical trial is currently being designed in order to validate the role of customizing treatment based on the plasma mRNA expression of BRCA1 and TS.

## Additional file

Additional file 1:
**Supplementary Methods.** **Table S1.** Mean mRNA levels in plasma and tumor. **Figure S1.** Original tumor and paired mouse xenografts. **(A)** The morphology of the original tumor diagnosed as adenocarcinoma with partial mucous cell carcinoma. **(B)** Paired xenografts in immunodeficient mice, confirmed by hematoxylin-eosin staining. There were no significant morphological differences between the original tumor sample and the paired xenografts in the mouse models. **Figure S2.** Validation of the stability of plasma mRNA. Plasma mRNA levels of all the genes were stable after being subjected to various conditions. **Figure S3.** Correlations between *in vitro* and *in vivo* inhibition rates for **(A)** docetaxel and **(B)** pemetrexed in the pilot study. **Figure S4.** Correlation between plasma and tumor mRNA expression of **(A)** BRCA1 (n = 32) and **(B)** TS (n = 40) in the pilot study. **Figure S5.** Correlations between mRNA expression levels and *in vitro* chemosensitivity in the pilot study. **(A)** Tumor BRCA1 and docetaxel sensitivity; **(B)** plasma BRCA1 and docetaxel sensitivity; **(C)** tumor TS and pemetrexed sensitivity; and **(D)** plasma TS and pemetrexed sensitivity. **Figure S6.** mRNA expression levels and *in vivo* chemosensitivity. Using a tumor inhibition cut-off of 50%, mouse models were divided into **(A)** docetaxel-sensitive and docetaxel-resistant subgroups and **(B)** pemetrexed-sensitive and pemetrexed-resistant subgroups. BRCA1 mRNA levels (dots) were higher in the docetaxel-sensitive group than in the docetaxel-resistant group (*P* < 0.001), and TS levels (dots) were higher in the pemetrexed-resistant group than in the pemetrexed-sensitive group (*P* = 0.013).
