# Supplementary material for: Plasma mRNA expression levels of BRCA1 and TS as potential predictive biomarkers for chemotherapy in gastric cancer
Source: J Transl Med. 2014 Dec 14;12:355. doi: 10.1186/s12967-014-0355-2 (PMC4302091; doi:10.1186/s12967-014-0355-2)

**Plasma mRNA expression levels of BRCA1 and TS as potential predictive biomarkers for chemotherapy in gastric cancer**

**Jie Shen, Jia Wei, Wenxian Guan, Hao Wang, Yitao Ding, Xiaoping Qian, Lixia Yu, Zhengyun Zou, Li Xie, Carlota Costa, Trever Bivona, Rafael Rosell, Baorui Liu**

**^*^** Jie Shen and Jia Wei contributed equally to this work

**Supplementary Appendix**

**Supplementary Methods**

**Study design**

For the pilot study of 40 of the 150 patients, each tumor sample was divided into three parts. For each tumor sample, sensitivity to docetaxel and pemetrexed was examined *in vitro* by histoculture drug response assay (HDRA)^1,2^ and *in vivo* by xenografts in immunodeficient mouse models. The remaining third of the tumor sample was formalin-fixed and paraffin-embedded (FFPE) for gene expression analysis. Gloves were worn during the entire process. Diagnosis of gastric cancer was confirmed by histopathology.

**HDRA for *in vitro* testing of chemosensitivity**

HDRA procedures were performed as reported by Furukawa and colleagues with slight modification^1, 2^. After histoculture, 100 μl of Hank’s balanced salt solution containing 0.1 mg/ml type I collagenase (Sigma, Shanghai, China) and 100 ul of 3-(4,5-Dimethyl-2-thiazotyl)-2,5-diphenyl-2H- tetrazoliumbromide (MTT) solution dissolved in 5 mg/ml phosphate buffer solution were added to each culture well and incubated for another 16 hours. After extraction with dimethyl sulfoxide, absorbance of the solution in each well was read at 540 nm. Absorbance per gram of cultured tumor tissue was calculated from the mean absorbance of tissue from 4 culture wells, and the tumor-tissue weight was determined before culture. The HDRA was regarded as evaluable when the mean absorbance of extracted formazan at 540 nm of the control tumor was 15 or more per gram. When the inhibition rate of the drug was a negative value, it was regarded as zero, which meant a complete lack of chemosensitivity.

We made an *a priori* decision to classify all the samples into sensitive and resistant groups using the minimum P value method modified by Lausen and Schumacher^3, 4^. Based on this method, tumors were defined as sensitive to docetaxel if the inhibition rate was ≥34.50% and as resistant if the inhibition rate was <34.50%, compared to untreated controls. Tumors were defined as sensitive to pemetrexed if the inhibition rate was ≥43.60% and as resistant if the inhibition rate was <43.60%, compared to untreated controls.

**Mouse xenograft models for *in vivo* testing of chemosensitivity**

Four- to six-week-old male BALB/c nude mice were housed in a barrier facility and acclimated to 12-hour light-dark cycles for at least two days before use. Each freshly-removed surgical tumor tissue was cut into several pieces of 3×3×3mm^3^ in a sterile Petri dish containing RPMI 1640 medium and 1% penicillin/streptomycin. Under anesthesia with isofluorane, fresh tumor pieces were transplanted within 30 mins to the 9-18 athymic immunodeficient mice by a small incision and subcutaneous pocket made in the dorsal flank in which one tumor piece was deposited^5^. While the pocket was still open, one drop of 1% penicillin/streptomycin solution was placed into the opening. Mice were observed daily for tumor growth. Eleven panels of immunodeficient mice with human-derived xenografts were successfully established from the 40 surgical specimens in the pilot study.

Doses and schedules of docetaxel and pemetrexed were chosen according to previous experience in animal experiments and represent the maximum tolerated or efficient doses. The injection volume was 0.2 ml/20 g body weight. The body weight of mice was determined every 2 days and the change in body weight was set as a variable for tolerability.

**mRNA expression in plasma and tumor**

***Total RNA extraction from plasma***

Two milliliters of blood were collected in EDTA tubes before surgery. They were immediately centrifuged at 3000g for 10 min at 4°C and recentrifuged at 12000g for 10 min at 4°C. 750 μl of plasma was carefully separated and mixed with 2250 μl TRIzol LS reagent (Invitrogen, [Carlsbad, C](http://en.wikipedia.org/wiki/Carlsbad,_California)A, USA) thoroughly, and stored at -80°C until use^6^. Each plasma-TRIzol LS mixture was thawed and mixed with 600 μl of chloroform. The RNA lysate was separated into three phases by centrifugation at 14000g for 15min at 4°C. The upper layer with RNA was transferred to new RNase-free tubes and further purified with a column named RNA pure link mini kit (Ambion, [Carlsbad, C](http://en.wikipedia.org/wiki/Carlsbad,_California)A, USA) according to the manufacturer’s instructions, dissolved in 35 μl of RNase-free water, and treated with DNase I (Ambion, [Carlsbad, C](http://en.wikipedia.org/wiki/Carlsbad,_California)A, USA).

***Total RNA extraction from FFPE tumor tissues***

Fresh gastric cancer specimens were placed in formalin immediately after surgery; after 24 hours, they were made into FFPE blocks. In the pilot study, the mouse xenografts were also made into FFPE blocks for gene expression analysis. Seven 7-μm sections were prepared from primary tumor blocks that contained at least 80% tumor cells. After hematoxylin-eosin staining, the cancerous parts were microdissected and transferred into a microcentrifuge tube. RNA was isolated in accordance with a proprietary procedure (European patent number EP1945764-B1). Briefly, paraffin was removed by xylene, and microdissected cancerous parts were lysed in a proteinase K-containing buffer at 60ºC for 16 h. RNA was purified by phenol and chloroform extractions followed by precipitation with isopropanol in the presence of sodium acetate at -20ºC. The RNA pellet was washed in 70% ethanol and resuspended in 53 μl of RNase-free water, followed by treatment with DNase I (Ambion, [Carlsbad, C](http://en.wikipedia.org/wiki/Carlsbad,_California)A, USA).

***Quantitative RT-PCR (qRT-PCR) assessment of gene expression***

M-MLV Reverse Transcriptase Kit (Invitrogen, [Carlsbad, C](http://en.wikipedia.org/wiki/Carlsbad,_California)A, USA) was used to generate cDNA for Q-PCR to detect the expression of β-actin (ACTB – used as endogenous control), BRCA1 and TS in the pilot study (40 patients) and the total cohort. Total RNA 1 μg was used for each RT reaction. Template cDNA was amplified with specific primers and probes for ACTB, BRCA1 and TS using Taqman Universal Master Mix (Applied Biosystems, Foster City, CA, USA).

The primers and probes were follows: ACTB (NM_001101.3) forward 5´ TGAGCGCGGCTACAGCTT 3´, reverse 5´ TCCTTAATGTCACGCACGATTT 3´, and probe 6FAM -5´ACCACCA CGGCCGAGCGG 3´ TAMRA; BRCA1 (NM_007294) forward 5´GGCTATCCTCTCAGAGTGACATTTTA 3´, reverse 5´GCTTTATCAGGTTATGTTGCATGGT 3´, and probe 6FAM -5´CCACTCAGCAGAGGG 3´ MGB. Primers and probes for TS were obtained from Applied Biosystems (Foster City, CA, USA): TS Assay IDs: Hs00426586_m1.

qRT-PCR was performed to quantify gene expression using the ABI Prism 7900HT Sequence Detection System (Applied Biosystems, Foster City, CA, USA). The PCR conditions were 50ºC for 2 min, 95ºC for 15 min, followed by 40 cycles at 95ºC for 15 s and 60ºC for 1 min.

**References**

1. Furukawa T, Kubota T, Hoffman RM. Clinical applications of the histoculture drug response assay. Clin Cancer Res. 1995; **1**(3): 305-11.

2. Hayashi Y, Kuriyama H, Umezu H, et al. Class III beta-tubulin expression in tumor cells is correlated with resistance to docetaxel in patients with completely resected non-small-cell lung cancer. Intern Med. 2009; **48**(4): 203-8.

3. Lausen B, Schumacher M. Maximally selected rank statistics. Biometrics*,* 1992; **48,** 73–85.

4. Hothorn T, Zeileis A. Generalized maximally selected statistics. Biometrics. 2008; **64**(4): 1263-9.

5. Fichtner I, Rolff J, Soong R, et al. Establishment of patient-derived non-small cell lung cancer xenografts as models for the identification of predictive biomarkers. Clin Cancer Res. 2008; **14**(20): 6456-68.

6. Chan RW, Wong J, Chan HL, et al. Aberrant concentrations of liver-derived plasma albumin mRNA in liver pathologies. Clin Chem. 2010; **56**(1): 82-9.

**Supplementary Table 1.** Mean mRNA levels in plasma and tumor

| Characteristic | All Patients N=150 N (%) | Mean BRCA1 mRNA in plasma (range) | Mean BRCA1 mRNA in tumor (range) | Mean TS mRNA in plasma (range) | Mean TS mRNA in tumor (range) |
| --- | --- | --- | --- | --- | --- |
| Age, y median | 64 (29-84) | 1.06 | 7.58 | 1.22 | 9.53 |
|  |  | (0.05-3.77) | (0.24-30.80) | (0.02-7.25) | (0.13-46.55) |
| Sex |  |  |  |  |  |
| Male | 110 (73.3%) | 1.14 | 7.77 | 1.33 | 9.83 |
| Female | 40 (26.7%) | 0.80 | 7.05 | 1.03 | 8.89 |
| Tumor Site |  |  |  |  |  |
| Distal | 57 (34.0%) | 1.04 | 8.19 | 1.40 | 9.42 |
| Proximal | 62 (41.3%) | 1.03 | 6.82 | 1.00 | 9.53 |
| Whole stomach | 37 (24.7%) | 1.05 | 7.98 | 1.29 | 9.86 |
| Stage |  |  |  |  |  |
| I | 17 (11.3%) | 0.85 | 5.72 | 1.16 | 6.97 |
| II | 34 (22.7%) | 1.17 | 8.48 | 1.20 | 9.80 |
| III | 94 (62.7%) | 1.00 | 7.41 | 1.23 | 9.41 |
| IV | 5 (3.3%) | 1.66 | 1.10 | 0.89 | 16.17 |
| Histological grade |  |  |  |  |  |
| 1 | 3 (2.0%) | 1.76 | 6.60 | 1.12 | 3.57 |
| 2 | 31 (20.7%) | 1.31 | 1.04 | 0.93 | 8.26 |
| 3 | 71 (47.3%) | 0.90 | 6.51 | 1.12 | 9.66 |
| Mixed 1–2 | 2 (1.3%) | 0.34 | 5.99 | 2.02 | 2.43 |
| Mixed 2–3 | 43 (28.7%) | 1.02 | 7.37 | 1.55 | 1.09 |
| Lymph node metastasis | |  |  |  |  |
| No | 35 (23.3%) | 1.04 | 7.50 | 1.20 | 9.09 |
| Yes | 115 (76.7%) | 1.04 | 7.60 | 1.27 | 9.71 |

There was no significant association between clinical characteristics and the mRNA expression levels of BRCA1 or TS (P>0.05).

**Supplementary Figure 1.** Original tumor and paired mouse xenografts. **(A)** The morphology of the original tumor diagnosed as adenocarcinoma with partial mucous cell carcinoma. **(B)** Paired xenografts in immunodeficient mice, confirmed by hematoxylin-eosin staining. There were no significant morphological differences between the original tumor sample and the paired xenografts in the mouse models.


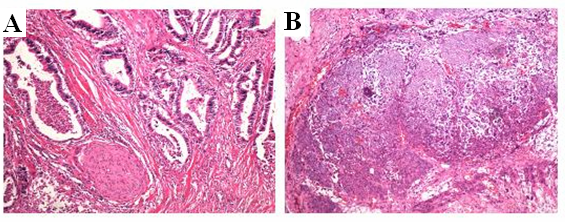


**Supplementary Figure 2.** Validation of the stability of plasma mRNA. Plasma mRNA levels of all the genes were stable after being subjected to various conditions.

**Sample1:**

**
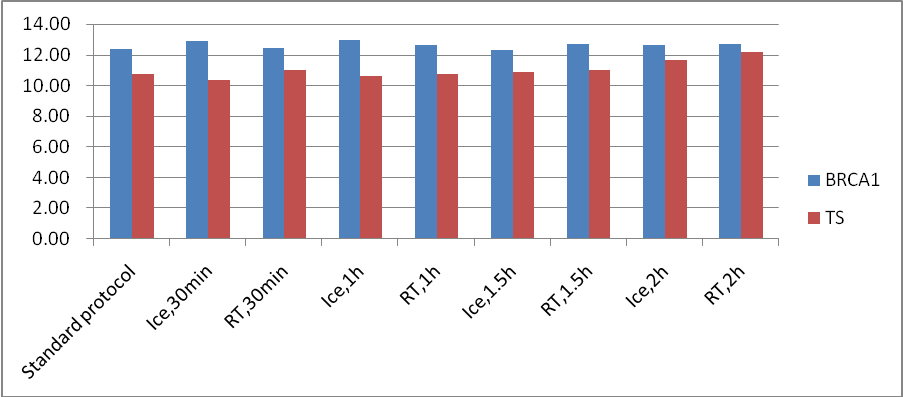
**

| Sample1 | Gene | Mean | STDEV | Coef Variation |
| --- | --- | --- | --- | --- |
|  | BRCA1 | 12.62 | 0.23 | 0.02 |
|  | TS | 11.02 | 0.57 | 0.05 |

**Sample2:**

**
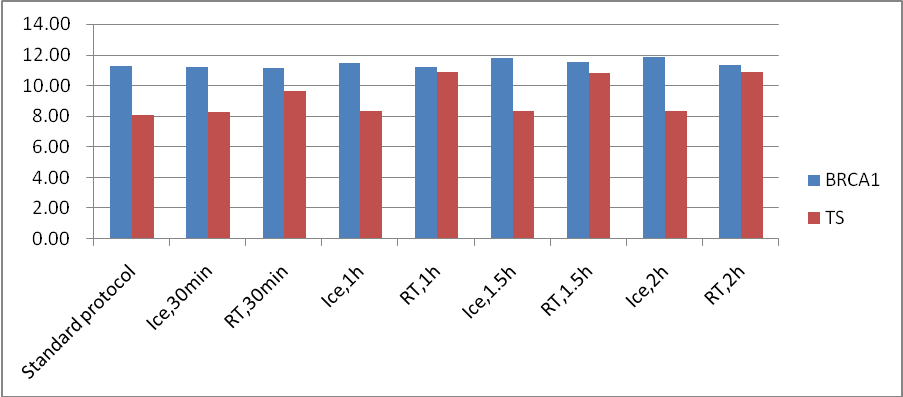
**

| Sample2 | Gene | Mean | STDEV | Coef Variation |
| --- | --- | --- | --- | --- |
|  | BRCA1 | 11.41 | 0.26 | 0.02 |
|  | TS | 9.29 | 1.27 | 0.14 |

**Sample3:**

**
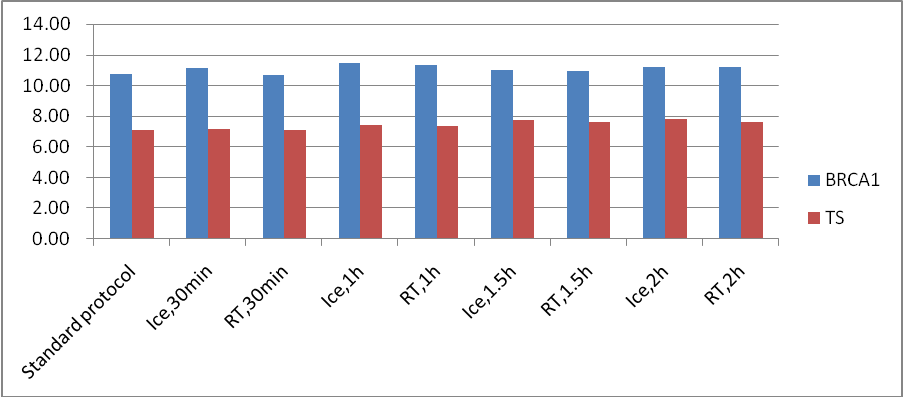
**

| Sample3 | gene | Mean | STDEV | Coef Variation |
| --- | --- | --- | --- | --- |
|  | BRCA1 | 11.07 | 0.27 | 0.02 |
|  | TS | 7.43 | 0.26 | 0.04 |

**Supplementary Figure 3.** Correlations between *in vitro* and *in vivo* inhibition rates for **(A)** docetaxel and **(B)** pemetrexed in the pilot study


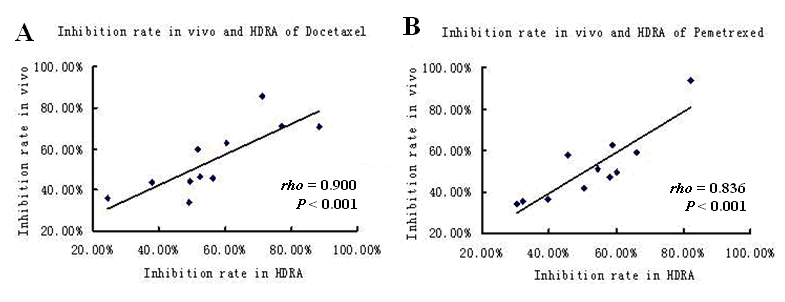


**Supplementary Figure 4.** Correlation between plasma and tumor mRNA expression of **(A)** BRCA1 (n=32) and **(B)** TS (n=40) in the pilot study


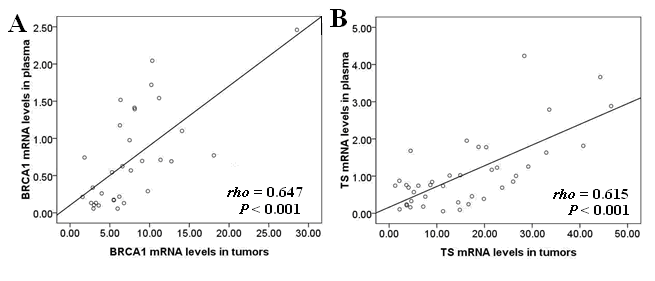


**Supplementary Figure 5.** Correlations between mRNA expression levels and *in vitro* chemosensitivity in the pilot study. **(A)** Tumor BRCA1 and docetaxel sensitivity; **(B)** plasma BRCA1 and docetaxel sensitivity; **(C)** tumor TS and pemetrexed sensitivity; and **(D)** plasma TS and pemetrexed sensitivity


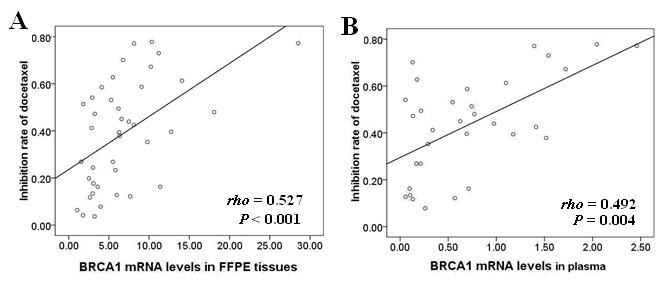


**
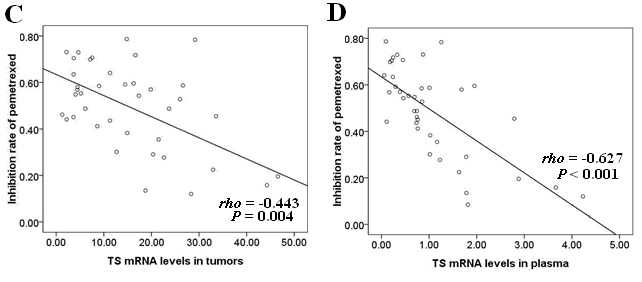
**

**Supplementary Figure 6.** mRNA expression levels and *in vivo* chemosensitivity. Using a tumor inhibition cut-off of 50%, mouse models were divided into **(A)** docetaxel-sensitive and docetaxel-resistant subgroups and **(B)** pemetrexed-sensitive and pemetrexed-resistant subgroups. BRCA1 mRNA levels (dots) were higher in the docetaxel-sensitive group than in the docetaxel-resistant group (*P* < 0.001), and TS levels (dots) were higher in the pemetrexed-resistant group than in the pemetrexed-sensitive group (*P* = 0.013).


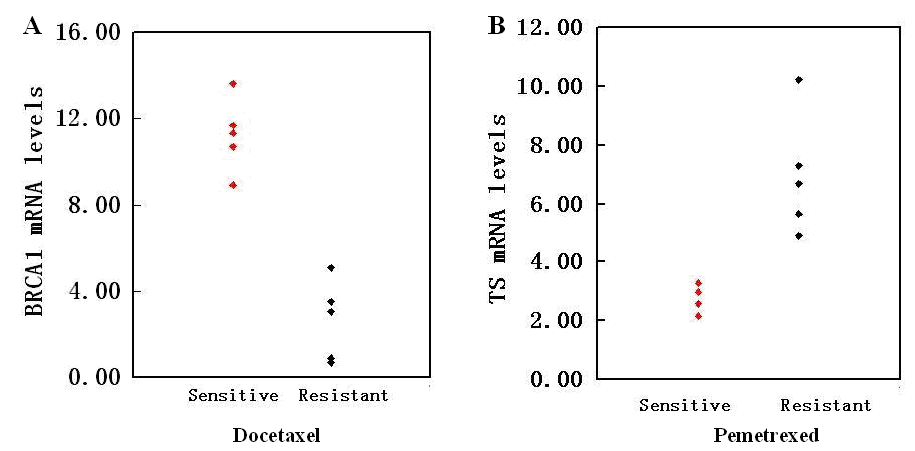

Supplement: Additional file 1: — Supplementary Methods. Table S1. Mean mRNA levels in plasma and tumor. Figure S1. Original tumor and paired mouse xenografts. (A) The morphology of the original tumor diagnosed as adenocarcinoma with partial mucous cell carcinoma. (B) Paired xenografts in immunodeficient mice, confirmed by hematoxylin-eosin staining. There were no significant morphological differences between the original tumor sample and the paired xenografts in the mouse models. Figure S2. Validation of the stability of plasma mRNA. Plasma mRNA levels of all the genes were stable after being subjected to various conditions. Figure S3. Correlations between in vitro and in vivo inhibition rates for (A) docetaxel and (B) pemetrexed in the pilot study. Figure S4. Correlation between plasma and tumor mRNA expression of (A) BRCA1 (n = 32) and (B) TS (n = 40) in the pilot study. Figure S5. Correlations between mRNA expression levels and in vitro chemosensitivity in the pilot study. (A) Tumor BRCA1 and docetaxel sensitivity; (B) plasma BRCA1 and docetaxel sensitivity; (C) tumor TS and pemetrexed sensitivity; and (D) plasma TS and pemetrexed sensitivity. Figure S6. mRNA expression levels and in vivo chemosensitivity. Using a tumor inhibition cut-off of 50%, mouse models were divided into (A) docetaxel-sensitive and docetaxel-resistant subgroups and (B) pemetrexed-sensitive and pemetrexed-resistant subgroups. BRCA1 mRNA levels (dots) were higher in the docetaxel-sensitive group than in the docetaxel-resistant group (P < 0.001), and TS levels (dots) were higher in the pemetrexed-resistant group than in the pemetrexed-sensitive group (P = 0.013). [file 12967_2014_355_MOESM1_ESM.doc]
